# Factor analysis of acute kidney injury in patients administered liposomal amphotericin B in a real-world clinical setting in Japan

**DOI:** 10.1038/s41598-020-72135-y

**Published:** 2020-09-14

**Authors:** Takahiro Takazono, Masato Tashiro, Yuki Ota, Yoko Obata, Tomotaro Wakamura, Taiga Miyazaki, Tomoya Nishino, Koichi Izumikawa

**Affiliations:** 1 Department of Infectious Diseases, Nagasaki University Graduate School of Biomedical Sciences, 1-7-1 Sakamoto, Nagasaki, 852-8501 Japan; 2 Department of Respiratory Medicine, Nagasaki University Hospital, Nagasaki, Japan; 3 Nagasaki University Infection Control and Education Center, Nagasaki University Hospital, Nagasaki, Japan; 4 Department of Nephrology, Nagasaki University Hospital, Nagasaki, Japan; 5 Medical Affairs Division, Sumitomo Dainippon Pharma Co., Ltd., Tokyo, Japan

**Keywords:** Fungal infection, Acute kidney injury

## Abstract

Liposomal amphotericin B (L-AMB) is a broad-spectrum antifungal drug that is used to treat fungal infections. However, clinical evidence of its use in patients with renal failure is limited. Here, we aimed to identify factors associated with acute kidney injury (AKI) in patients administered L-AMB. We retrospectively utilized a combination of Diagnosis Procedure Combination data and laboratory data obtained from hospitals throughout Japan between April 2008 and January 2018. In total, 507 patients administered L-AMB were identified. After L-AMB treatment initiation, AKI, which was defined as a ≥ 1.5-fold increase within 7 days or ≥ 0.3 mg/dL increase within 2 days in serum creatinine according to the KDIGO criteria, was recognized in 37% of the total patients (189/507). The stages of AKI were stage 1 in 20%, stage 2 in 11%, and stage 3 in 7%. Five factors were associated with AKI of all stages: prior treatment with angiotensin-converting enzyme inhibitors/angiotensin-receptor blockers or carbapenem; concomitant administration of catecholamines or immunosuppressants; and ≥ 3.52 mg/kg/day of L-AMB dosing. Serum potassium < 3.5 mEq/L before L-AMB therapy was associated with severe AKI of stage 2 and 3. Altogether, these factors should be carefully considered to reduce the occurrence of AKI in patients administered L-AMB.

## Introduction

Invasive fungal infections frequently occur in immunocompromised patients and critically ill patients and are associated with high rates of morbidity and mortality^1–5^. Amphotericin B is a broad-spectrum antifungal drug that covers clinically relevant yeasts and molds that cause mycosis, such as aspergillosis, candidiasis, cryptococcosis, and mucormycosis^6^. However, the use of amphotericin B has been limited because of its high incidence of toxicity, such as nephrotoxicity, liver disorder, or hypokalemia^6^. Liposomal amphotericin B (L-AMB), which encapsulates amphotericin B in a liposomal membrane, was developed to reduce the toxicity of amphotericin B without reducing its antifungal activity^6^. This specific liposomal formulation reduces tissue distribution to kidneys and drug-associated nephrotoxicity^7,8^. However, regardless of the reduced nephrotoxicity, physicians are reluctant to prescribe L-AMB, especially for patients with renal failure, because of its association with the occurrence of acute kidney injury (AKI)^9,10^. Identifying the factors associated with the development of AKI in patients receiving L-AMB may thus help to promote its appropriate usage and reduce the occurrence of AKI.

Several studies conducted in single facilities have reported the factors associated with AKI in patients administered L-AMB. Yamazaki et al. demonstrated that pre-existing renal dysfunction and the concomitant use of nephrotoxic or antifungal drugs are associated with AKI during L-AMB treatment^9^. They defined AKI as an increase in serum creatinine to levels above 0.3 mg/dL or 1.5-fold higher than those at baseline. Saito et al. identified that the high level of creatinine clearance, which is defined as Grade 1 according to the Common Terminology Criteria for Adverse Events (CTCAE) standard, at the start of L-AMB treatment and concomitant drug treatment are the factors associated with the occurrence of decreased renal function^10^. However, large-scale clinical evidence of AKI-associated factors in patients administered L-AMB in Japan is limited. Therefore, based on a combination of Diagnosis Procedure Combination (DPC) data and laboratory data obtained from hospitals throughout Japan, we defined AKI as a ≥ 1.5-fold increase within 7 days or ≥ 0.3 mg/dL increase within 2 days in serum creatinine levels and sought to examine the factors associated with AKI in patients treated with L-AMB.

## Results

### Characteristics of patients administered L-AMB

**Figure 1:**
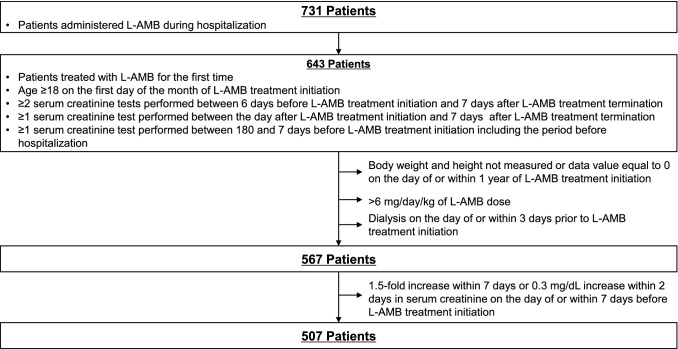
Selection of the study population. *L-AMB* liposomal amphotericin B.

By applying the inclusion and exclusion criteria and based on the definitions described in “Methods”, we identified 507 patients administered L-AMB treatment throughout Japan (Fig. 1). Only patients treated in public or private hospitals containing ≥ 200 beds were included in the analysis; thus, patients treated in university hospitals were excluded. The patient characteristics of the study population are shown in Table 1. A total of 332 cases (65%) were male patients, and 175 cases (35%) were female patients. Mean age was 66 years-old, mean body mass index (BMI) was 21.5 ± 3.7 kg/m^2^, and mean baseline estimated glomerular filtration rate (eGFR) was 96.1 ± 46.2 mL/min. More than half of the examined patients (63%, 320/507) were administered L-AMB in the hematology department. Fungal infections were observed in 36% (184/507) of patients, with aspergillosis as the most common mycosis. The median period of L-AMB administration was 11 days and median daily dose was 2.5 mg/kg/day (Table 2).

**Table 1:** Characteristics of patients in the study population.

Patient characteristics	Cases (%) (N = 507)	Mean ± SD
**Sex**		
Male	332 (65%)	
Female	175 (35%)	
**Age (years)**		66 ± 15
< 65	191 (38%)	
≥ 65	316 (62%)	
18–29	14 (3%)	
30–39	28 (6%)	
40–49	33 (7%)	
50–59	52 (10%)	
60–69	145 (29%)	
70–79	153 (30%)	
80–89	75 (15%)	
≥ 90	7 (1%)	
**BMI (kg/m**^**2**^**)**		21.5 ± 3.7
< 18.5	102 (20%)	
≥ 18.5 to < 25	328 (65%)	
≥ 25	77 (15%)	
**eGFR at baseline (mL/min)**		96.1 ± 46.2
≥ 90	235 (46%)	
≥ 60 to < 90	191 (38%)	
≥ 45 to < 60	52 (10%)	
≥ 30 to < 45	19 (4%)	
≥ 15 to < 30	9 (2%)	
< 15	1 (0%)	
**Treatment department**		
Hematology	320 (63%)	
Internal medicine (except for hematology)	146 (29%)	
Surgical	38 (7%)	
Other	3 (1%)	
**Diagnosis**		
Fungal infection	184 (36%)	
Aspergillosis	128 (25%)	
Candidiasis	35 (7%)	
Cryptococcosis	13 (3%)	
Zygomycosis	5 (1%)	
Aspergillosis, candidiasis	1 (0%)	
Aspergillosis, cryptococcosis	1 (0%)	
Aspergillosis*,* candidiasis*,* cryptococcosis	1 (0%)	
Neutropenia	51 (10%)	
Other	157 (31%)	
Unknown	115 (23%)	

*BMI* body mass index, *eGFR* estimated glomerular filtration rate, *SD* standard deviation.

**Table 2:** Duration and dose of L-AMB treatment.

L-AMB treatment	Cases (%) (N = 507)	Median
**Administration duration (days)**		11
< 7	147 (29%)	
≥ 7 to < 14	145 (29%)	
≥ 14 to < 21	97 (19%)	
≥ 21 to < 28	50 (10%)	
≥ 28	68 (13%)	
**Mean daily dose (mg/kg/day)**		2.5
< 2	110 (22%)	
≥ 2 to < 2.5	147 (29%)	
≥ 2.5 to < 3	144 (28%)	
≥ 3	106 (21%)	

*L-AMB* liposomal amphotericin B.

### Occurrence of acute kidney injury (AKI) in patients administered L-AMB

To evaluate the occurrence of AKI in patients after L-AMB therapy initiation, we defined the occurrence of AKI as a ≥ 1.5-fold increase within 7 days or ≥ 0.3 mg/dL increase within 2 days in serum creatinine levels between the day after L-AMB treatment initiation and 7 days following treatment termination (Fig. S1). As shown in Table 3, the overall occurrence of AKI was 37% (189/507). AKI patients were assigned stages 1–3 according to the KDIGO AKI criteria (stage 1: ≥ 1.5 to < twofold or ≥ 0.3 mg/dL creatinine increase, stage 2: ≥ 2 to < threefold creatinine increase, stage 3: ≥ threefold creatinine increase or ≥ 4.0 mg/dL of creatinine or the initiation of dialysis) between the date of the minimum creatinine reference measurement for AKI and 7 days following L-AMB treatment termination (the maximum period was 89 days). The stages of AKI were stage 1 in 20%, stage 2 in 11%, and stage 3 in 7%. Among patients with AKI stage 3, 2 cases required dialysis. AKI occurred at approximately 9 days after the start of L-AMB administration in all patients. For 2 patients requiring dialysis, treatment began 20 days after L-AMB therapy initiation.

**Table 3:** AKI in patients administered L-AMB.

Occurrence of AKI	Cases (%) (N = 507)
**AKI**	
Total	189 (37%)
Cr ≥ 1.5-fold within 7 days	175 (35%)
∆Cr ≥ 0.3 mg/dL within 2 days	97 (19%)
**AKI stage**	
Stage 1 (Cr ≥ 1.5 to < twofold or ∆Cr ≥ 0.3 mg/dL)	100 (20%)
Stage 2 (Cr ≥ 2 to < threefold)	55 (11%)
Stage 3 (Cr ≥ threefold or Cr ≥ 4.0 mg/dL or dialysis)	34 (7%)
Dialysis	2 (0%)

Period from L-AMB therapy initiation to AKI	Days
**All patients**	9.3 ± 10.4 (189 cases)
Patients with AKI except for dialysis	9.2 ± 10.4 (187 cases)
Patients with dialysis	
Time to Cr ≥ 1.5 or ∆Cr ≥ 0.3 mg/dL	18.0 ± 1.0 (2 cases)
Time to dialysis	20.0 ± 0.0 (2 cases)

*AKI* acute kidney injury, *L-AMB* liposomal amphotericin B.

### Identification of factors associated with the occurrence of AKI in patients receiving L-AMB

To identify the factors associated with AKI in patients administered L-AMB, we evaluated 62 patient characteristics, including sex, age, disease history, patient condition before and during L-AMB treatment, prior and concomitant use of drugs, and L-AMB dosing and period. By performing univariate regression analysis with the 62 patient characteristics, we identified 27 candidate factors associated with AKI (*p* < 0.2) (Table 4). These included < 65 years old, disease history (hypertension, heart failure), baseline patient conditions (catecholamine therapy, ≥ 60 mL/min eGFR, and < 3.5 mEq/L potassium), patient conditions during L-AMB administration (catecholamine treatment and < 3.5 serum potassium), prior treatment with immunosuppressants, steroids, angiotensin-converting enzyme (ACE) inhibitors/angiotensin-receptor blockers (ARBs), diuretics, several antibiotics, and cytotoxic antineoplastic agents, concomitant treatment with immunosuppressants, steroids, ACE inhibitors/ARBs, diuretics, several antibiotics, and ≥ 1,000 mL/day of fluid replacement, and the daily dose of L-AMB.

**Table 4:** Univariate regression analysis of the factors associated with AKI in patients administered L-AMB.

Variables	Odds ratio (95% CI)	P-value
**Sex/age**		
Female	0.982 (0.655–1.472)	0.929
Age ≥ 65	**0.764 (0.515–1.135)**	**0.182**
**Disease history**		
Diabetes	1.266 (0.849–1.886)	0.247
Diabetes treated with insulin	1.295 (0.817–2.052)	0.271
Hypertension	**1.385 (0.933–2.056)**	**0.106**
Chronic kidney disease	1.859 (0.703–4.914)	0.211
Hepatic dysfunction^a^	1.069 (0.594–1.922)	0.825
Heart failure	**1.525 (0.948–2.453)**	**0.082**
**Patient condition prior to L-AMB treatment**		
Catecholamine treatment^b^	**1.667 (0.774–3.589)**	**0.192**
Albumin ≤ 3 g/dL	1.150 (0.720–1.836)	0.558
eGFR < 60 mL/min	**0.664 (0.382–1.154)**	**0.146**
Potassium < 3.5 mEq/L	**1.446 (0.941–2.222)**	**0.093**
Potassium > 5.0 mEq/L	0.801 (0.238–2.702)	0.721
**Patient condition after the initiation of L-AMB treatment**		
Catecholamine treatment^b^	**3.109 (1.651–5.855)**	**< 0.001**
Potassium < 3.5 mEq/L	**1.329 (0.899–1.966)**	**0.154**
**Therapy before L-AMB treatment**		
NSAIDs	1.079 (0.722–1.613)	0.711
Immunosuppressants	**1.703 (1.077–2.694)**	**0.023**
Steroids	**1.454 (0.956–2.212)**	**0.080**
Contrast agents (iodine)	1.287 (0.863–1.919)	0.216
ACE inhibitors/ARBs	**2.049 (1.155–3.635)**	**0.014**
Diuretics	**1.341 (0.912–1.972)**	**0.135**
Aminoglycosides	0.961 (0.571–1.618)	0.881
Fourth generation cephem	**1.611 (1.095–2.371)**	**0.016**
Other cephem (except for fourth generation)	1.127 (0.757–1.680)	0.556
Trimethoprims	**1.514 (1.029–2.227)**	**0.035**
Injected fluoroquinolones	1.169 (0.764–1.789)	0.472
Oral fluoroquinolones	0.909 (0.557–1.483)	0.701
Sulbactam	1.108 (0.693–1.771)	0.669
Tazobactam	0.986 (0.650–1.497)	0.948
Carbapenem	**2.435 (1.608–3.689)**	**< 0.001**
Teicoplanin	**1.687 (0.987–2.883)**	**0.056**
Vancomycin	**1.759 (1.176–2.631)**	**0.006**
Polymyxin B sulfate	**1.637 (0.967–2.770)**	**0.066**
Other antimicrobials	1.093 (0.710–1.682)	0.688
Cytotoxic chemotherapy	**1.390 (0.947–2.042)**	**0.093**
Non-cytotoxic chemotherapy	1.143 (0.646–2.022)	0.647
Fluid replacement (≥ 1,000 mL/day)	1.265 (0.862–1.855)	0.230
**Concomitant treatment with L-AMB therapy**		
NSAIDs	1.169 (0.755–1.810)	0.484
Immunosuppressants	**3.053 (1.532–6.084)**	**0.002**
Steroids	**1.619 (1.058–2.477)**	**0.026**
Contrast agents (iodine)	1.021 (0.588–1.771)	0.941
ACE inhibitors/ARBs	**1.698 (0.897–3.213)**	**0.104**
Diuretics	**1.573 (1.070–2.312)**	**0.021**
Aminoglycosides	1.059 (0.630–1.781)	0.828
Fourth generation cephem	0.915 (0.527–1.591)	0.754
Other cephem (except for fourth generation)	0.853 (0.521–1.397)	0.528
Trimethoprims	1.170 (0.790–1.732)	0.432
Injected fluoroquinolones	1.238 (0.807–1.899)	0.328
Oral fluoroquinolones	1.255 (0.538–2.929)	0.600
Sulbactam	1.292 (0.675–2.473)	0.439
Tazobactam	1.034 (0.663–1.614)	0.883
Carbapenem	1.063 (0.723–1.562)	0.757
Teicoplanin	**2.196 (1.277–3.778)**	**0.004**
Vancomycin	**1.662 (1.103–2.505)**	**0.015**
Polymyxin B sulfate	1.538 (0.705–3.356)	0.280
Other antimicrobials	1.219 (0.781–1.904)	0.383
Cytotoxic chemotherapy	0.991 (0.572–1.715)	0.973
Non-cytotoxic chemotherapy	0.742 (0.296–1.858)	0.523
Fluid replacement (≥ 1,000 mL/day)	**1.662 (1.107–2.497)**	**0.014**
**L-AMB administration**		
Mean daily dose	**1.340 (1.057–1.697)**	**0.016**
Treatment duration	1.006 (0.993–1.019)	0.363
Cumulative dose	1.002 (0.998–1.007)	0.306

Bold font indicates p < 0.2 variables. N = 442 for hepatic dysfunction, N = 371 for albumin ≤ 3 g/dL prior to L-AMB treatment, N = 444 for other variables.

*ACE* angiotensin-converting enzyme, *ARBs* angiotensin-receptor blockers, *CI* confidence interval, *eGFR* estimated glomerular filtration rate, *L-AMB* liposomal amphotericin B, *NSAIDs* nonsteroidal anti-inflammatory drugs.

^a^≥ 120 IU/L of aspartate transaminase (AST) or alanine transaminase (ALT).

^b^Catecholamine treatment was defined as the state of shock.

By using stepwise regression, 7 of the 27 variables identified by univariate logistic regression were selected. In addition to the 7 variables, 2 clinically important variables (eGFR ≥ 60 mL/min and serum potassium < 3.5 mEq/L prior to L-AMB therapy), which were identified by univariate logistic regression (*p* < 0.2), were simultaneously evaluated in the final multivariate regression model. As shown in Table 5, five independent factors that were significantly associated with AKI in patients treated with L-AMB (*p* < 0.05) were identified through multivariate regression analysis. These factors included: catecholamine treatment during L-AMB administration (odds ratio [OR] 2.315; 95% confidence interval [CI] 1.173–4.569), treatment with ACE inhibitors/ARBs (OR 2.070; 95% CI 1.120–3.824) or carbapenem (OR 2.034; 95% CI 1.303–3.176) prior to L-AMB administration, concomitant treatment with immunosuppressants (OR 2.248; 95% CI 1.083–4.663), and ≥ 3.52 mg/kg/day of L-AMB dose (OR 2.648; 95% CI 1.302–5.386).

**Table 5:** Multivariate regression analysis of the factors related to AKI in patients administered L-AMB.

Variables	Odds ratio (95% CI)	P-value	VIF
**Patient condition prior to L-AMB treatment**			
eGFR < 60 mL/min	0.734 (0.407–1.325)	0.305	1.029
Potassium (K) < 3.5 mEq/L	1.165 (0.731–1.858)	0.520	1.034
**Patient condition after the initiation of L-AMB treatment**			
Catecholamine treatment^a^	**2.315 (1.173–4.569)**	**0.016**	**1.040**
Treatment before L-AMB therapy			
ACE inhibitors/ARBs	**2.070 (1.120–3.824)**	**0.020**	**1.023**
Carbapenem	**2.034 (1.303–3.176)**	**0.002**	**1.057**
**Concomitant treatment with L-AMB therapy**			
Immunosuppressants	**2.248 (1.083–4.663)**	**0.030**	**1.029**
Teicoplanin	1.595 (0.880–2.891)	0.124	1.071
Vancomycin	1.540 (0.986–2.405)	0.058	1.062
**L-AMB administration**			
Mean daily dose, ≥ 3.52 mg/kg/day	**2.648 (1.302–5.386)**	**0.007**	**1.038**

Bold font indicates statistically significant variables (p < 0.05). N = 444.

*ACE* angiotensin-converting enzyme, *ARBs* angiotensin-receptor blockers, *CI* confidence interval, *eGFR* estimated glomerular filtration rate, *L-AMB*, liposomal amphotericin B.

^a^Catecholamine treatment was defined as the state of shock.

Sensitivity analysis revealed that treatment with carbapenem prior to L-AMB administration (OR 1.747; 95% CI 1.036–2.946) and concomitant treatment with immunosuppressants (OR 2.699; 95% CI 1.183–6.155) were associated with AKI in patients with stage 1 AKI (Tables S1, S2). On the other hand, serum potassium < 3.5 mEq/L prior to L-AMB therapy (OR 1.828; 95% CI 1.007–3.319), catecholamine treatment during L-AMB administration (OR 2.442; 95% CI 1.056–5.645), treatment with ACE inhibitors/ARBs (OR 2.511; 95% CI 1.109–5.687) or carbapenem (OR 3.033; 95% CI 1.626–5.654) prior to L-AMB administration, and ≥ 2.93 mg/kg/day of L-AMB dose (OR 2.425; 95% CI 1.319–4.458) were associated with stages 2 and 3 AKI (Tables S3, S4).

## Discussion

L-AMB is a broad-spectrum antifungal drug that is used for the treatment of invasive fungal infections. However, clinical evidence of its use in patients with renal failure is limited. In this study, we aimed to identify factors associated with the occurrence of AKI in patients administered L-AMB. Based on a combination of claims data and laboratory data, we identified the following five AKI-associated factors: prior treatment with ACE inhibitors/ARBs or carbapenem, concomitant administration of catecholamines or immunosuppressants, and ≥ 3.52 mg/kg/day of L-AMB dosing. Moreover, serum potassium < 3.5 mEq/L before L-AMB therapy was associated with severe AKI of stage 2 and 3. Therefore, patients with those factors might require frequently monitoring for AKI. As our dataset consisted of many patients (507 patients) administered L-AMB, and was obtained from hospitals throughout Japan, the factors identified in this study could serve as reliable indicators.

Several studies have reported an association between factors identified in this study and the occurrence of AKI. First, immunosuppressant calcineurin inhibitors are known to increase the risk of renal injury^11^. In the present study, we found that calcineurin inhibitors, such as tacrolimus (58%; 26/45 patients) and cyclosporin (29%; 13/45 patients), were frequently administered to this patient cohort. A careful adjustment of tacrolimus dose and monitoring of drug concentration during L-AMB treatment could thus ameliorate the occurrence of AKI. Second, consistent with our finding that the use of catecholamines during L-AMB administration was associated with AKI, patients with shock treated with catecholamines often causes renal failure^12^. Third, ACE inhibitors/ARBs may increase serum creatinine owing to the decrease in intraglomerular pressure, resulting in an increase in the occurrence of renal dysfunction, especially when the deterioration of general conditions, such as infection, occurs^13^. Furthermore, carbapenems, such as meropenem and imipenem/cilastatin, have been reported to cause an increase in serum creatinine and serum urea^14^. As imipenem is highly nephrotoxic, it is used in combination with the nephrotoxicity-reducing drug cilastatin. However, in this study, as the occurrence of AKI was high in patients treated with meropenem (44%, 91/206 patients) and imipenem/cilastatin (54%, 47/87 patients) prior to L-AMB therapy initiation compared to all patients (37%), those drugs may be used for critically ill patients.

We showed that ≥ 3.52 mg/kg/day of L-AMB doses is significantly associated with the development of AKI. L-AMB may induce AKI through tubular injury and renal vasoconstriction^15,16^. Tubular injury may be induced by intramembranous pore formation or vacuolation of the epithelial cells in the distal convoluted tubule^17^, while renal vascular resistance may be increased by activating the tubuloglomerular feedback mechanism^18^. Previous studies with a small population did not identify a correlation between L-AMB dosage and the occurrence of AKI^9,10^. Since the present study is based on data retrieved from a large patient cohort, our results might be more reliable than those obtained with a small cohort. Altogether, L-AMB daily dose and the cumulative dose should be carefully considered.

In the present study, serum potassium levels < 3.5 mEq/L (hypokalemia) prior to L-AMB treatment were associated with stages 2 and 3 AKI. Importantly, chronic, persistent hypokalemia is associated with AKI through vacuolar degeneration of proximal renal tubule cells and distal renal tubule cells^19^. This degeneration may be caused by impaired angiogenesis^20^, tubular cytoplasmic vacuolization^21^, and/or interstitial scarring caused by renal cytogenesis^22^. Therefore, intervention for hypokalemia prior to L-AMB administration is essential for reducing the occurrence of AKI.

Baseline eGFR was found to be an important indicator of renal function. Importantly, baseline eGFR levels did not correlate with the duration and daily dose of L-AMB administration (*p* = 0.414 and *p* = 0.387, respectively, the Jonckheere–Terpstra trend test). These results suggest that L-AMB could be administered without dosage adjustment.

During L-AMB treatment, patients frequently showed abnormal serum levels of blood components, such as hematopoietic cells or electrolytes. Therefore, we opted to focus on patients with serum levels of blood components within the range of the CTCAE standard on the day of and within 7 days before L-AMB treatment initiation, and examined those levels between the day after L-AMB treatment initiation and 7 days following treatment termination. By investigating blood cell parameters (WBC and PLT) and the levels of serum biochemicals (total protein, BUN, uric acid, AST, ALT, LDH, γ-GTP, ALP, total bilirubin, glucose, Ca, Na, K, and Cl), we observed abnormal decreases below or increases over the standard values for these parameters in 41–78% of patients administered L-AMB. Severe abnormalities, as defined by a CTCAE grade ≥ 3 except for death, were observed in 2–33% of patients administered L-AMB. Notably, lower baseline eGFR was correlated with a higher occurrence of abnormal serum levels of chloride (p = 0.045, the Cochran-Armitage trend test).

In this study, we identified 2 patients who underwent dialysis after the completion of L-AMB treatment. Those patients died immediately after the completion of dialysis without renal recovery. One patient received dialysis for 2 days started from 6 days after L-AMB therapy termination and died on the day after completion of dialysis, while another patient received dialysis for 3 days initiated from 5 days after L-AMB therapy termination and died 4 days after completion of dialysis. In these patients, serum creatinine levels on the day before death did not recover to the levels observed before AKI (data not shown).

This study had several limitations owing to its retrospective nature. First, the generalizability of the findings presented herein requires further discussion. This is because the database used to analyze the current data did not contain data from university hospitals where infectious disease experts may work and patients with severe infectious disease and comorbidities may visit for treatment, and facilities that had less than 200 beds. Moreover, hospital transfers of patients could not be tracked. Thus, our study findings may not be fully representative of patients treated throughout Japan. Second, we could not evaluate AKI by assessing decreased urine volume and/or AKI biomarkers^23^, as these parameters could not be obtained from the database used in this study. Therefore, further large-scale prospective studies, which include the value of serum creatinine as well as urine volume and AKI biomarkers, are required to verify the results. Finally, for the sensitivity analysis performed to identify the factors related to stages 2 and 3 AKI, although more variables (two additional variable) than the permissible number (8) were identified, all factors were included in the multivariate regression model to adjust all important confounding factors.

## Conclusions

Based on a combination of claims data and laboratory data obtained throughout Japan, we identified five AKI-related factors: prior treatment with ACE inhibitors/ARBs or carbapenem, concomitant administration of catecholamines or immunosuppressants, and ≥ 3.52 mg/kg/day of L-AMB dosing. Moreover, serum potassium < 3.5 mEq/L before L-AMB therapy was associated with severe AKI in patients with stages 2 and 3 AKI. Therefore, those factors should be carefully considered to attenuate the development of AKI in patients administered L-AMB.

## Methods

### Study design

This was a retrospective, multicenter, observational study based on data obtained between April 2008 and January 2018; data were obtained from the electronic medical information database of Medical Data Vision Co., Ltd (MDV). Although L-AMB was launched in June 2006 in Japan, MDV contains data from April 2008. Therefore, we analyzed the data for the maximum period at the initiation of this study. This contained the DPC hospital data, medical fee reimbursement claims, and clinical laboratory test data from 345 facilities in Japan. In addition to claims and abstract discharge data, the database contains the following information for each patient: age, sex, primary diagnosis, comorbidities at admission and the post-admission complications coded according to the International Classification of Diseases, 10th revision codes written in Japanese, medical procedures, including types of surgery, coded with original Japanese codes, daily records of drug administration and devices used, length of hospital stay, and discharge status^24^. The dates of hospital admission, surgery, bedside procedures, drugs administered, and discharge were recorded using a uniform data submission format^24^. This study was conducted in accordance with the Declaration of Helsinki. The data analyzed in the present study were anonymously processed by the database provider in accordance with the Act on the Protection of Personal Information of Japan and other related regulations. For the usage of unlinkable de-identified data, ethical approval and informed consent were waived, according to the Japanese Ethical Guidelines for Medical and Health Research Involving Human Subjects by the Ministry of Education, Culture, Sports, Science, and Technology and the Ministry of Health, Labour, and Welfare of Japan. The study received ethical approval from Nagasaki University School of Medicine Research Ethics Committee (Approval Number 18033038-4). The STROBE statement checklist was included in Supplementary Table S5.

### Study population

Patients administered L-AMB during hospitalization were selected according to a set of criteria. The inclusion criteria were: L-AMB treatment for the first time, age ≥ 18 years-old on the first day of the month of L-AMB treatment initiation, at least two measurements of serum creatinine performed between 6 days before L-AMB treatment initiation and 7 days after L-AMB treatment termination, at least one measurement of serum creatinine performed between the day after L-AMB treatment initiation and 7 days after L-AMB treatment termination, and at least one measurement of serum creatinine performed between 180 and 7 days before L-AMB treatment initiation including the period before hospitalization. The exclusion criteria were: unavailability of height or weight data on the day of or within 1 year of L-AMB treatment initiation, which were required to calculate eGFR, a L-AMB dose > 6 mg/kg/day, dialysis on the day of or within 3 days prior to L-AMB administration initiation, and ≥ 1.5-fold increase within 7 days or ≥ 0.3 mg/dL increase within 2 days in serum creatinine on the day of or within 7 days before L-AMB treatment initiation to exclude patients who developed AKI before the initiation of L-AMB therapy.

### Assessments

L-AMB treatment duration was defined as the time from treatment initiation to discontinuation. L-AMB discontinuation was defined as an administration interval ≥ 8 days. According to the KDIGO AKI criteria^25^, we defined AKI as a ≥ 1.5-fold increase within 7 days or ≥ 0.3 mg/dL increase within 2 days in serum creatinine between the day after L-AMB treatment initiation and 7 days following L-AMB administration termination (Fig. S1). As the time for a creatinine measurement in a day could not be obtained from database, ≥ 1.5-fold or ≥ 0.3 mg/dL increases in serum creatinine were evaluated for a total of 8 or 3 days, respectively. Urine volume was not employed as part of the definition because we could not obtain this information from the database. AKI patients were assigned three stages based on the KDIGO AKI criteria between the date of the minimum creatinine reference measurement for AKI and 7 days following L-AMB treatment termination (the maximum period was 89 days). The criteria were: stage 1, ≥ 1.5- to < 2-fold increase or ≥ 0.3 mg/dL increase in serum creatinine; stage 2, ≥ 2- to < 3-fold increase in serum creatinine; stage 3, ≥ threefold increase in serum creatinine, ≥ 4.0 mg/dL of serum creatinine or the initiation of dialysis^25^. Adjusted eGFR was calculated by using the following formula for Japanese which correlates with other formulas such as CKD-EPI and MDRD^26^:

$$ {\text{Adjusted }}\;{\text{eGFR }}\left( {{\text{mL}}/{\min}} \right) \, = \, [{194 } \times {\text{ Cr }}\left( {{\text{mg}}/{\text{dL}}} \right)^{{{-}{1}.0{94}}} \times {\text{ age }}\left( {{\text{years}}} \right)^{{{-}0.{287}}} \left( { \times \, 0.{739 }\;{\text{for }}\;{\text{women}}} \right) \, /{ 1}.{\text{73 m}}^{{2}} ] \times {\text{ Body}}\;{\text{ surface}}\;{\text{ area }}\left( {{\text{m}}^{{2}} } \right) $$

where, Body surface area (m^2^) = 0.007184 × [weight (kg)]^0.425^ × [height (cm)]^0.725^, Cr = the concentration of serum creatinine (mg/dL).

For calculation of adjusted eGFR at baseline, baseline serum creatinine was defined as the minimum level of serum creatinine measured between 180 and 7 day before the initiation of L-AMB therapy, including the period before hospitalization^27^. eGFR levels were divided into six groups: ≥ 90 mL/min; ≥ 60 to < 90 mL/min; ≥ 45 to < 60 mL/min; ≥ 30 to < 45 mL/min; ≥ 15 to < 30 mL/min; and < 15 mL/min. To examine the abnormality of blood components during L-AMB treatment, we opted to focus on patients with serum levels of blood components within the range of the CTCAE standard on the day of and within 7 days before L-AMB treatment initiation, and evaluated the serum levels of blood cell parameters (WBC and PLT) and the levels of serum biochemicals (total protein, BUN, uric acid, AST, ALT, LDH, γ-GTP, ALP, total bilirubin, glucose, Ca, Na, K, and Cl) between the day after L-AMB treatment initiation and 7 days following treatment termination. Abnormal serum levels were defined as a decrease and increase in the above and below standard values, respectively, while severe abnormality was defined as a Grade ≥ 3 CTCAE, except for those of deceased patients. The missing values, including baseline serum creatinine, were not used and complemented for analysis.

### Variables and statistical analyses

Logistic regression analysis was conducted to identify the factors associated with AKI in patients administered L-AMB. The clinicians selected 62 variables from the patient characteristics that were considered to be related to AKI. Sex and age were obtained on the first day of the month of L-AMB therapy initiation. Diabetes, hypertension, chronic kidney disease, and heart failure were identified using the corresponding ICD-10 codes which were registered on the month of L-AMB therapy initiation. The ICD-10 codes for chronic kidney disease are summarized in Supplementary Table S6. Treatment with insulin, which was performed for patients with diabetes, was identified between the admission date and the date of L-AMB therapy initiation. Hepatic dysfunction was defined as ≥ 120 IU/L of either Aspartate transaminase (AST) or alanine transaminase (ALT) on the most recent day of L-AMB therapy initiation measured between the day of and 7 days before L-AMB initiation. Treatment with catecholamine prior to L-AMB therapy was identified on the day of or within 7 days before L-AMB therapy initiation, while concomitant catecholamine treatment during L-AMB therapy was identified between the day after L-AMB therapy initiation and the date of L-AMB therapy termination. Hypoalbuminemia (serum albumin ≤ 3 g/dL), hypokalemia (serum potassium < 3.5 mEq/L), and hyperkalemia (serum potassium > 5.0 mEq/L) before L-AMB therapy were evaluated by using the most recent value of serum albumin and potassium on the date of L-AMB therapy initiation measured between the day of and 7 days before L-AMB therapy initiation. On the other hands, hypokalemia after the initiation of L-AMB therapy was evaluated with the minimum value of serum potassium measured between the day after L-AMB therapy initiation and the date of L-AMB therapy termination. Drug treatment before L-AMB therapy was identified between the admission date and the day before L-AMB therapy initiation. Concomitant drug treatment during L-AMB therapy was identified between the date of L-AMB therapy initiation and termination. Fluid replacement (≥ 1,000 mL/day) before L-AMB treatment initiation was identified within 7 days before L-AMB therapy initiation, while that after L-AMB therapy initiation was identified between the date of L-AMB therapy initiation and 6 days after L-AMB therapy initiation or the date of L-AMB therapy termination. We selected patients who had no missing values for variables with a P value < 0.2 in univariate logistic regression analysis conducted with the maximum number of patients. 62 variables in selected patients were then subjected to univariate logistic regression analysis. Variables with a P value < 0.2 were selected using stepwise regression according to the backward elimination selection algorithms using the Akaike information criterion (AIC). In addition to the factors selected by stepwise regression, the clinically important 4 factors: baseline eGFR < 60 mL/min, serum potassium < 3.5 mEq/L prior to L-AMB therapy, treatment with contrast agents prior to L-AMB therapy, and concomitant treatment with non-steroidal anti-inflammatory drugs (NSAIDs) were included in the final multivariate logistic regression model when those variables had a P value < 0.2 in the univariate logistic regression analysis. Multivariate logistic regression analysis was thus conducted with the above variables. The variance inflation factor (VIF) was calculated to identify the multicollinearity of the explanatory variables. Receiver operating characteristic (ROC) curves were used to identify the cut-off values for continuous variables, including the daily and cumulative doses of L-AMB. For sensitivity analysis, patients without AKI and with stage 1 AKI or those without AKI and with stages 2 and 3 AKI were subjected to logistic regression analysis. Continuous variables are presented as average ± standard deviation. Student’s t-test was employed to compare two groups for continuous variables, while the Fisher’s exact test was used for two categorical variables. The correlation between baseline eGFR and treatment duration and daily dose of L-AMB was evaluated by the Jonckheere–Terpstra trend test, while that between baseline eGFR and abnormality of blood components between the day after L-AMB treatment initiation and 7 days following treatment termination was evaluated by the Cochran–Armitage trend test.

## Supplementary information

Supplementary Information.
