# Supplementary material for: Factor analysis of acute kidney injury in patients administered liposomal amphotericin B in a real-world clinical setting in Japan
Source: Sci Rep. 2020 Sep 14;10:15033. doi: 10.1038/s41598-020-72135-y (PMC7490360; doi:10.1038/s41598-020-72135-y)
Supplement: Supplementary file 1 — Supplementary Information. [file 41598_2020_72135_MOESM1_ESM.docx]

**Supplementary information**

**Factor analysis of acute kidney injury in patients administered liposomal amphotericin B in a real-world clinical setting in Japan**

Takahiro Takazono^1,2*^, Masato Tashiro^1,3^, Yuki Ota^4^, Yoko Obata^4^, Tomotaro Wakamura^5^, Taiga Miyazaki^1,2^, Tomoya Nishino^4^, and Koichi Izumikawa^1,3^

*^1^Department of Infectious Diseases, Nagasaki University Graduate School of Biomedical Sciences, Nagasaki, Japan*

*^2^Department of Respiratory Medicine, Nagasaki University Hospital, Nagasaki, Japan*

*^3^Nagasaki University Infection Control and Education Center, Nagasaki University Hospital, Nagasaki, Japan*

*^4^Department of Nephrology, Nagasaki University Hospital,* *Nagasaki, Japan*

*^5^Medical Affairs Division, Sumitomo Dainippon Pharma* Co., Ltd. *Tokyo, Japan*

***Correspondence:** Takahiro TAKAZONO, M.D., Ph.D.; Department of Infectious Diseases, Nagasaki University Graduate School of Biomedical Sciences, 1-7-1 Sakamoto, Nagasaki 852-8501, Japan

Phone: +81-95-819-7273; Fax: +81-95-849-7285

E-mail: takahiro-takazono@nagasaki-u.ac.jp

**Keywords:** Liposomal amphotericin B; Acute kidney injury, Retrospective study, Logistic regression analysis

**Running title:** Acute kidney injury-related factors during L-AMB therapy

**Supplementary Figure 1.** Acute kidney injury **(**AKI) definition


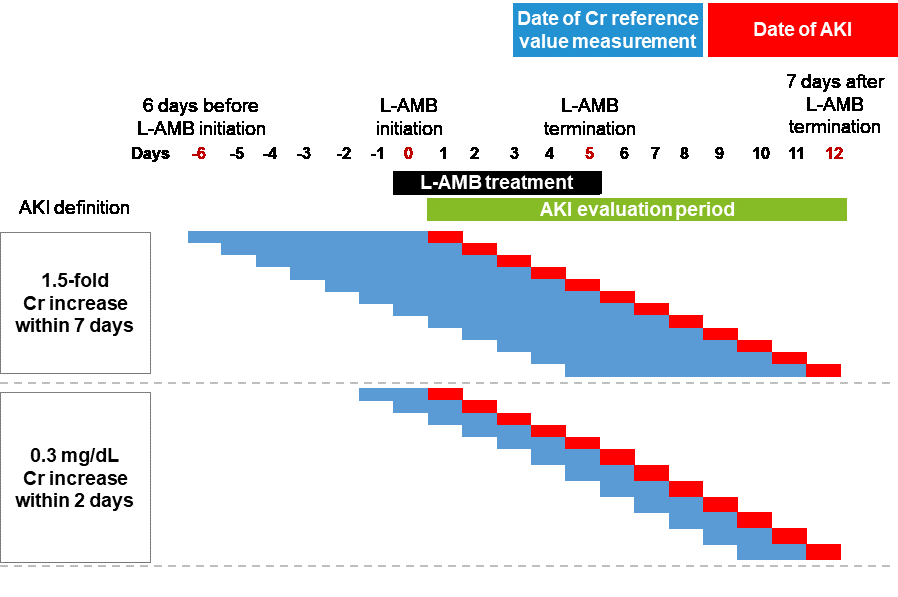


AKI, acute kidney injury; Cr, creatinine; L-AMB, liposomal amphotericin B. We thank for the kind permission to use this image prduced by Akinori Takahashi (Deloitte Tohmatsu Consulting LLC).

**Supplementary Table 1.** Univariate regression analysis of the factors associated with AKI stage 1 in patients administered L-AMB.

| **Variables** | **Odds Ratio (95% CI)** | **P-value** |
| --- | --- | --- |
| Sex/age |  |  |
| Female | 0.816 (0.484-1.375) | 0.445 |
| Age ≥65 | 0.960 (0.580-1.589) | 0.874 |
| Disease history |  |  |
| Diabetes | 1.350 (0.822-2.217) | 0.235 |
| Diabetes treated with insulin | 1.198 (0.670-2.142) | 0.542 |
| Hypertension | 1.130 (0.685-1.867) | 0.632 |
| **Chronic kidney disease** | **2.433 (0.820-7.215)** | **0.109** |
| Hepatic dysfunction*^a^* | 1.425 (0.722-2.812) | 0.307 |
| **Heart failure** | **1.554 (0.867-2.785)** | **0.139** |
| Patient condition prior to L-AMB treatment | |  |
| **Catecholamine treatment^b^** | **1.857 (0.752-4.587)** | **0.180** |
| Albumin ≤3 g/dL | 0.959 (0.544-1.689) | 0.885 |
| eGFR <60 mL/min | 0.816 (0.419-1.589) | 0.550 |
| Potassium <3.5 mEq/L | 1.161 (0.669-2.016) | 0.596 |
| Potassium >5.0 mEq/L | 0.773 (0.161-3.711) | 0.748 |
| Patient condition after the initiation of L-AMB treatment | |  |
| **Catecholamine treatment^b^** | **2.620 (1.217-5.640)** | **0.014** |
| Potassium <3.5 mEq/L | 1.106 (0.681-1.798) | 0.683 |
| Therapy before L-AMB treatment |  |  |
| NSAIDs | 0.923 (0.552-1.543) | 0.761 |
| Immunosuppressants | 1.264 (0.698-2.291) | 0.439 |
| Steroids | 1.218 (0.727-2.042) | 0.453 |
| Contrast agents (iodine) | 1.130 (0.682-1.874) | 0.634 |
| ACE inhibitors/ARBs | 1.573 (0.754-3.279) | 0.227 |
| Diuretics | 1.183 (0.730-1.915) | 0.495 |
| Aminoglycosides | 1.131 (0.603-2.121) | 0.702 |
| Fourth generation cephem | 1.281 (0.789-2.079) | 0.317 |
| Other cephem (except for fourth generation) | 1.326 (0.810-2.170) | 0.262 |
| **Trimethoprims** | **1.446 (0.892-2.344)** | **0.134** |
| Injected fluoroquinolones | 1.242 (0.733-2.103) | 0.420 |
| Oral fluoroquinolones | 0.837 (0.446-1.572) | 0.580 |
| Sulbactam | 1.248 (0.704-2.212) | 0.448 |
| Tazobactam | 1.131 (0.677-1.891) | 0.638 |
| **Carbapenem** | **1.879 (1.131-3.121)** | **0.015** |
| **Teicoplanin** | **1.681 (0.873-3.236)** | **0.120** |
| **Vancomycin** | **1.582 (0.957-2.616)** | **0.074** |
| Polymyxin B sulfate | 1.450 (0.748-2.811) | 0.271 |
| Other antimicrobials | 0.953 (0.548-1.657) | 0.865 |
| Cytotoxic chemotherapy | 1.231 (0.761-1.991) | 0.397 |
| **Non-cytotoxic chemotherapy** | **1.623 (0.845-3.117)** | **0.146** |
| **Fluid replacement (≥1000 mL/day)** | **1.479 (0.912-2.400)** | **0.113** |
| Concomitant treatment with L-AMB therapy | |  |
| NSAIDs | 0.988 (0.563-1.734) | 0.966 |
| **Immunosuppressants** | **3.219 (1.450-7.146)** | **0.004** |
| **Steroids** | **1.461 (0.860-2.484)** | **0.161** |
| Contrast agents (iodine) | 0.796 (0.379-1.672) | 0.547 |
| ACE inhibitors/ARBs | 1.205 (0.514-2.825) | 0.668 |
| Diuretics | 1.189 (0.735-1.925) | 0.481 |
| Aminoglycosides | 0.931 (0.475-1.825) | 0.835 |
| Fourth generation cephem | 0.836 (0.409-1.709) | 0.623 |
| Other cephem (except for fourth generation) | 0.885 (0.477-1.640) | 0.698 |
| Trimethoprims | 1.302 (0.799-2.121) | 0.290 |
| Injected fluoroquinolones | 1.179 (0.689-2.018) | 0.548 |
| Oral fluoroquinolones | 0.709 (0.197-2.546) | 0.598 |
| **Sulbactam** | **1.892 (0.914-3.915)** | **0.086** |
| Tazobactam | 1.051 (0.602-1.832) | 0.862 |
| Carbapenem | 1.054 (0.650-1.709) | 0.831 |
| **Teicoplanin** | **2.259 (1.181-4.323)** | **0.014** |
| **Vancomycin** | **1.497 (0.897-2.501)** | **0.123** |
| Polymyxin B sulfate | 1.605 (0.626-4.112) | 0.324 |
| Other antimicrobials | 1.309 (0.756-2.267) | 0.336 |
| Cytotoxic chemotherapy | 0.603 (0.270-1.344) | 0.216 |
| Non-cytotoxic chemotherapy | 0.822 (0.266-2.545) | 0.734 |
| **Fluid replacement (≥1000 mL/day)** | **1.796 (1.065-3.026)** | **0.028** |
| L-AMB administration |  |  |
| Mean daily dose | 1.036 (0.753-1.425) | 0.830 |
| Treatment duration | 1.003 (0.987-1.019) | 0.735 |
| Cumulative dose | 1.001 (0.995-1.006) | 0.809 |

ACE, angiotensin-converting enzyme; ARBs, angiotensin-receptor blockers; CI, confidence interval; eGFR, estimated glomerular filtration rate; L-AMB, liposomal amphotericin B; NSAIDs, nonsteroidal anti-inflammatory drugs. Bold font indicates p<0.2 variables. N=361 for hepatic dysfunction, N=304 for albumin ≤3 g/dL prior to L-AMB treatment, N=362 for other variables.

*^a^* ≥120 IU/L of aspartate transaminase (AST) or alanine transaminase (ALT).

*^b^*Catecholamine treatment was defined as the state of shock.

**Supplementary Table 2.** Multivariate regression analysis of the factors related to AKI stage 1 in patients administered L-AMB.

| **Variables** | **Odds Ratio (95% CI)** | **P-value** | **VIF** |
| --- | --- | --- | --- |
| Disease history |  |  |  |
| Chronic kidney disease | 2.803 (0.905-8.677) | 0.074 | 1.019 |
| Patient condition after the initiation of L-AMB treatment | |  |  |
| Catecholamine treatment*^a^* | 2.039 (0.916-4.537) | 0.081 | 1.031 |
| Treatment before L-AMB therapy |  |  |  |
| **Carbapenem** | **1.747 (1.036-2.946)** | **0.036** | **1.010** |
| Concomitant treatment with L-AMB therapy | |  |  |
| **Immunosuppressants** | **2.699 (1.183-6.155)** | **0.018** | **1.015** |
| Fluid replacement (≥1000 mL/day) | 1.585 (0.917-2.741) | 0.099 | 1.052 |

CI, confidence interval; L-AMB, liposomal amphotericin B. Bold font indicates statistically significant variables (p<0.05). N=362.

*^a^*Catecholamine treatment was defined as the state of shock.

**Supplementary Table 3.** Univariate regression analysis of the factors associated with AKI stages 2 and 3 in patients administered L-AMB.

| **Variables** | **Odds Ratio (95% CI)** | **P-value** |
| --- | --- | --- |
| Sex/age |  |  |
| Female | 1.183 (0.709-1.973) | 0.520 |
| **Age ≥65** | **0.605 (0.367-0.998)** | **0.049** |
| Disease history |  |  |
| Diabetes | 1.179 (0.705-1.975) | 0.530 |
| Diabetes treated with insulin | 1.403 (0.787-2.501) | 0.252 |
| **Hypertension** | **1.708 (1.035-2.820)** | **0.036** |
| Chronic kidney disease | 1.263 (0.327-4.873) | 0.735 |
| Hepatic dysfunction*^a^* | 0.712 (0.302-1.681) | 0.439 |
| **Heart failure** | **1.495 (0.818-2.732)** | **0.191** |
| Patient condition prior to L-AMB treatment | |  |
| Catecholamine treatment^b^ | 1.466 (0.545-3.946) | 0.449 |
| Albumin ≤3 g/dL | 1.443 (0.760-2.739) | 0.263 |
| **eGFR <60 mL/min** | **0.509 (0.230-1.125)** | **0.095** |
| **Potassium <3.5 mEq/L** | **1.795 (1.054-3.057)** | **0.031** |
| Potassium >5.0 mEq/L | 0.831 (0.173-3.994) | 0.817 |
| Patient condition after the initiation of L-AMB treatment | |  |
| **Catecholamine treatment^b^** | **3.665 (1.759-7.638)** | **<0.001** |
| **Potassium <3.5 mEq/L** | **1.635 (0.973-2.746)** | **0.063** |
| Therapy before L-AMB treatment |  |  |
| NSAIDs | 1.266 (0.761-2.108) | 0.364 |
| **Immunosuppressants** | **2.254 (1.295-3.925)** | **0.004** |
| **Steroids** | **1.788 (1.011-3.162)** | **0.046** |
| **Contrast agents (iodine)** | **1.472 (0.887-2.444)** | **0.135** |
| **ACE inhibitors/ARBs** | **2.605 (1.328-5.111)** | **0.005** |
| **Diuretics** | **1.540 (0.931-2.546)** | **0.093** |
| Aminoglycosides | 0.788 (0.387-1.605) | 0.512 |
| **Fourth generation cephem** | **2.064 (1.252-3.404)** | **0.005** |
| Other cephem (except for fourth generation) | 0.940 (0.557-1.587) | 0.817 |
| **Trimethoprims** | **1.589 (0.968-2.610)** | **0.067** |
| Injected fluoroquinolones | 1.094 (0.630-1.899) | 0.750 |
| Oral fluoroquinolones | 0.988 (0.530-1.840) | 0.969 |
| Sulbactam | 0.965 (0.519-1.796) | 0.911 |
| Tazobactam | 0.844 (0.486-1.466) | 0.547 |
| **Carbapenem** | **3.352 (1.869-6.013)** | **<0.001** |
| **Teicoplanin** | **1.693 (0.866-3.310)** | **0.124** |
| **Vancomycin** | **1.967 (1.182-3.272)** | **0.009** |
| **Polymyxin B sulfate** | **1.846 (0.970-3.513)** | **0.062** |
| Other antimicrobials | 1.254 (0.729-2.157) | 0.413 |
| **Cytotoxic chemotherapy** | **1.587 (0.963-2.614)** | **0.070** |
| Non-cytotoxic chemotherapy | 0.682 (0.290-1.604) | 0.380 |
| Fluid replacement (≥1000 mL/day) | 1.071 (0.653-1.754) | 0.787 |
| Concomitant treatment with L-AMB therapy | |  |
| NSAIDs | 1.382 (0.801-2.385) | 0.245 |
| **Immunosuppressants** | **2.877 (1.252-6.613)** | **0.013** |
| **Steroids** | **1.817 (1.028-3.212)** | **0.040** |
| Contrast agents (iodine) | 1.279 (0.655-2.498) | 0.472 |
| **ACE inhibitors/ARBs** | **2.270 (1.082-4.763)** | **0.030** |
| **Diuretics** | **2.143 (1.292-3.555)** | **0.003** |
| Aminoglycosides | 1.203 (0.629-2.298) | 0.576 |
| Fourth generation cephem | 1.003 (0.499-2.016) | 0.994 |
| Other cephem (except for fourth generation) | 0.820 (0.429-1.565) | 0.547 |
| Trimethoprims | 1.041 (0.626-1.733) | 0.877 |
| Injected fluoroquinolones | 1.303 (0.757-2.244) | 0.340 |
| **Oral fluoroquinolones** | **1.874 (0.722-4.865)** | **0.197** |
| Sulbactam | 0.709 (0.261-1.927) | 0.500 |
| Tazobactam | 1.017 (0.572-1.807) | 0.955 |
| Carbapenem | 1.072 (0.652-1.762) | 0.784 |
| **Teicoplanin** | **2.130 (1.088-4.169)** | **0.027** |
| **Vancomycin** | **1.854 (1.107-3.107)** | **0.019** |
| Polymyxin B sulfate | 1.466 (0.545-3.946) | 0.449 |
| Other antimicrobials | 1.126 (0.631-2.009) | 0.687 |
| Cytotoxic chemotherapy | 1.461 (0.768-2.778) | 0.248 |
| Non-cytotoxic chemotherapy | 0.656 (0.185-2.323) | 0.513 |
| **Fluid replacement (≥1000 mL/day)** | **1.535 (0.909-2.590)** | **0.109** |
| L-AMB administration |  |  |
| **Mean daily dose** | **1.685 (1.256-2.260)** | **<0.001** |
| Treatment duration | 1.010 (0.993-1.028) | 0.239 |
| **Cumulative dose** | **1.004 (0.999-1.010)** | **0.139** |

ACE, angiotensin-converting enzyme; ARBs, angiotensin-receptor blockers; CI, confidence interval; eGFR, estimated glomerular filtration rate; L-AMB, liposomal amphotericin B; NSAIDs, nonsteroidal anti-inflammatory drugs. Bold font indicates p<0.2 variables. N=354 for hepatic dysfunction, N=295 for albumin ≤3 g/dL prior to L-AMB treatment, N=356 for other variables.

*^a^*≥120 IU/L of aspartate transaminase (AST) or alanine transaminase (ALT).

*^b^*Catecholamine treatment was defined as the state of shock.

**Supplementary Table 4.** Multivariate regression analysis of the factors related to AKI stages 2 and 3 in patients administered L-AMB.

| **Variables** | **Odds Ratio (95% CI)** | **P-value** | **VIF** |
| --- | --- | --- | --- |
| Disease history |  |  |  |
| Hypertension | 1.665 (0.936-2.963) | 0.083 | 1.096 |
| Patient condition prior to L-AMB treatment |  |  |  |
| eGFR <60 mL/min | 0.502 (0.209-1.207) | 0.124 | 1.037 |
| **Potassium <3.5 mEq/L** | **1.828 (1.007-3.319)** | **0.047** | **1.046** |
| Patient condition after the initiation of L-AMB treatment | |  |  |
| **Catecholamine treatment*^a^*** | **2.442 (1.056-5.645)** | **0.037** | **1.033** |
| Treatment before L-AMB therapy |  |  |  |
| Contrast agents (iodine) | 1.270 (0.715-2.258) | 0.415 | 1.071 |
| **ACE inhibitors/ARBs** | **2.511 (1.109-5.687)** | **0.027** | **1.164** |
| Trimethoprims | 1.546 (0.873-2.739) | 0.135 | 1.113 |
| **Carbapenem** | **3.033 (1.626-5.654)** | **<0.001** | **1.033** |
| L-AMB administration |  |  |  |
| **Mean daily dose, ≥2.93 mg/kg/day** | **2.425 (1.319-4.458)** | **0.004** | **1.144** |
| Cumulative dose, ≥23.64 mg/kg | 1.787 (0.986-3.238) | 0.056 | 1.082 |

ACE, angiotensin-converting enzyme; ARBs, angiotensin-receptor blockers; CI, confidence interval; eGFR, estimated glomerular filtration rate; L-AMB, liposomal amphotericin B. Bold font indicates statistically significant variables (p<0.05). N=356.

*^a^*Catecholamine treatment was defined as the state of shock.

**Supplementary Table 5.** STROBE Statement

|  | Item No | Recommendation | Relevant text from manuscript |
| --- | --- | --- | --- |
| **Title and abstract** | 1 | (*a*) Indicate the study’s design with a commonly used term in the title or the abstract | Abstract |
|  |  | (*b*) Provide in the abstract an informative and balanced summary of what was done and what was found | Abstract |
| Introduction | | |  |
| Background/rationale | 2 | Explain the scientific background and rationale for the investigation being reported | Introduction, paragraph 1 |
| Objectives | 3 | State specific objectives, including any prespecified hypotheses | Introduction, paragraph 2 |
| Methods | | |  |
| Study design | 4 | Present key elements of study design early in the paper | Methods, subheading 1, paragraph 1 |
| Setting | 5 | Describe the setting, locations, and relevant dates, including periods of recruitment, exposure, follow-up, and data collection | Methods, subheading 1, paragraph 1 |
| Participants | 6 | (*a*) *Cohort study*—Give the eligibility criteria, and the sources and methods of selection of participants. Describe methods of follow-up  *Case-control study*—Give the eligibility criteria, and the sources and methods of case ascertainment and control selection. Give the rationale for the choice of cases and controls  *Cross-sectional study*—Give the eligibility criteria, and the sources and methods of selection of participants | Methods, subheading 2, paragraph 1 |
|  |  | (*b*) *Cohort study*—For matched studies, give matching criteria and number of exposed and unexposed  *Case-control study*—For matched studies, give matching criteria and the number of controls per case | n/a |
| Variables | 7 | Clearly define all outcomes, exposures, predictors, potential confounders, and effect modifiers. Give diagnostic criteria, if applicable | Methods, subheading 3, paragraph 1 |
| Data sources/ measurement | 8* | For each variable of interest, give sources of data and details of methods of assessment (measurement). Describe comparability of assessment methods if there is more than one group | Methods, subheading 4, paragraph 1 |
| Bias | 9 | Describe any efforts to address potential sources of bias | Methods, subheading 2, paragraph 1 |
| Study size | 10 | Explain how the study size was arrived at | Methods, subheading 1, paragraph 1 |
| Quantitative variables | 11 | Explain how quantitative variables were handled in the analyses. If applicable, describe which groupings were chosen and why | Methods, subheading 4, paragraph 1 |
| Statistical methods | 12 | (*a*) Describe all statistical methods, including those used to control for confounding | Methods, subheading 4, paragraph 1 |
|  |  | (*b*) Describe any methods used to examine subgroups and interactions | Methods, subheading 3, paragraph 1 |
|  |  | (*c*) Explain how missing data were addressed | Methods, subheading 3/4, paragraph 1 |
|  |  | (*d*) *Cohort study*—If applicable, explain how loss to follow-up was addressed  *Case-control study*—If applicable, explain how matching of cases and controls was addressed  *Cross-sectional study*—If applicable, describe analytical methods taking account of sampling strategy | n/a |
|  |  | (*e*) Describe any sensitivity analyses | Methods, subheading 4, paragraph 1 |

Continued on next page

| Results | | | Relevant text from manuscript |
| --- | --- | --- | --- |
| Participants | 13* | (a) Report numbers of individuals at each stage of study—eg numbers potentially eligible, examined for eligibility, confirmed eligible, included in the study, completing follow-up, and analysed | Figure 1 |
|  |  | (b) Give reasons for non-participation at each stage | n/a |
|  |  | (c) Consider use of a flow diagram | Figure 1 |
| Descriptive data | 14* | (a) Give characteristics of study participants (eg demographic, clinical, social) and information on exposures and potential confounders | Table 1 |
|  |  | (b) Indicate number of participants with missing data for each variable of interest | Table 4/5, Supplementary Table 1/2/3/4 |
|  |  | (c) *Cohort study*—Summarise follow-up time (eg, average and total amount) | n/a |
| Outcome data | 15* | *Cohort study*—Report numbers of outcome events or summary measures over time | n/a |
|  |  | *Case-control study—*Report numbers in each exposure category, or summary measures of exposure | Table 3, Results, subheading 2, paragraph 1 |
|  |  | *Cross-sectional study—*Report numbers of outcome events or summary measures | n/a |
| Main results | 16 | (*a*) Give unadjusted estimates and, if applicable, confounder-adjusted estimates and their precision (eg, 95% confidence interval). Make clear which confounders were adjusted for and why they were included | Results, subheading 2, paragraph 1, 2 and 3, Table 4/5, Supplementary Table 1/2/3/4 |
|  |  | (*b*) Report category boundaries when continuous variables were categorized | Results, subheading 2, paragraph 2 and 3, Table 5, Supplementary Table 4 |
|  |  | (*c*) If relevant, consider translating estimates of relative risk into absolute risk for a meaningful time period | n/a |
| Other analyses | 17 | Report other analyses done—eg analyses of subgroups and interactions, and sensitivity analyses | Results, subheading 2, paragraph 3, Supplementary Table 1/2/3/4 |
| Discussion | | |  |
| Key results | 18 | Summarise key results with reference to study objectives | Discussion, paragraph 1 |
| Limitations | 19 | Discuss limitations of the study, taking into account sources of potential bias or imprecision. Discuss both direction and magnitude of any potential bias | Discussion, paragraph 8 |
| Interpretation | 20 | Give a cautious overall interpretation of results considering objectives, limitations, multiplicity of analyses, results from similar studies, and other relevant evidence | Discussion, paragraph 8 |
| Generalisability | 21 | Discuss the generalisability (external validity) of the study results | Discussion, paragraph 8 |
| Other information | | |  |
| Funding | 22 | Give the source of funding and the role of the funders for the present study and, if applicable, for the original study on which the present article is based | Competing interests |

*Give information separately for cases and controls in case-control studies and, if applicable, for exposed and unexposed groups in cohort and cross-sectional studies.

**Note:** An Explanation and Elaboration article discusses each checklist item and gives methodological background and published examples of transparent reporting. The STROBE checklist is best used in conjunction with this article (freely available on the Web sites of PLoS Medicine at http://www.plosmedicine.org/, Annals of Internal Medicine at http://www.annals.org/, and Epidemiology at http://www.epidem.com/). Information on the STROBE Initiative is available at www.strobe-statement.org.

**Supplementary Table 6.** ICD-10 codes for chronic kidney disease.

| **ICD10 code** | **Disease name** |
| --- | --- |
| N18.1 | Chronic kidney disease, stage 1 |
| N18.2 | Chronic kidney disease, stage 2 |
| N18.3 | Chronic kidney disease, stage 3 |
| N18.4 | Chronic kidney disease, stage 4 |
| N18.5 | Chronic kidney disease, stage 5 |
| N18.9 | Chronic kidney disease, unspecified |
